# Orthodontic Elastic Separator-Induced Periodontal Abscess: A Case Report

**DOI:** 10.1155/2012/463903

**Published:** 2012-01-15

**Authors:** Talia Becker, Alex Neronov

**Affiliations:** ^1^Department of Oral Pathology and Oral Medicine, The Maurice & Gabriela Goldschleger School of Dental Medicine, Tel Aviv University, Tel Aviv, Israel; ^2^Israel Defense Forces, Medical Corps, Israel

## Abstract

*Aim*. Orthodontic elastic bands were proposed as being the source of gingival abscesses that can rapidly lead to bone loss and teeth exfoliation. We report an adolescent, otherwise, healthy patient whose periodontal status was sound. Shortly after undergoing preparations for orthodontic treatment consisting of orthodontic separators, he presented with a periodontal abscess for which there was no apparent etiology. A non-orthoradial X-ray was inconclusive, but an appropriate one revealed a subgingival orthodontic separator as the cause of the abscess. Removal of the separator and thorough scaling led to complete resolution of the abscess, but there was already residual mild damage to the alveolar bone. *Summary*. Failure to use appropriate imaging to reveal the cause of gingival abscesses can result in the delay of implementing treatment and halting irreversible alveolar bone loss. An inflammatory process restricted to the gingiva and refractive to conventional therapy should raise the possibility of a foreign body etiology.

## 1. Introduction

Local anatomic and iatrogenic factors may promote plaque retention and proliferation of microorganisms in the periodontal pocket, resulting in progressive inflammatory changes [1]. An inflammatory process restricted to the gingiva and refractive to conventional therapy should raise the possibility of a foreign body etiology [2]. Several reported cases of bone loss and teeth exfoliation were reported in association with orthodontic elastic bands [3–5], especially when they had been used to close a midline diastema between maxillary incisors. However, there are only a few reported cases of periodontal destruction caused by displaced orthodontic separators [6, 7]. Commonly employed therapeutic modalities include a combination of laser treatment, antibiotics, splinting, and orthodontics [8]. In order to avoid complications, it was recommended to use brightly colored elastic bands and to remove them after two weeks [9]. This report describes a case of a periodontal abscess associated with a displaced orthodontic separator and emphasizes the importance of appropriate X-rays for accurate diagnosis.

## 2. Case Report

**Figure 1:**
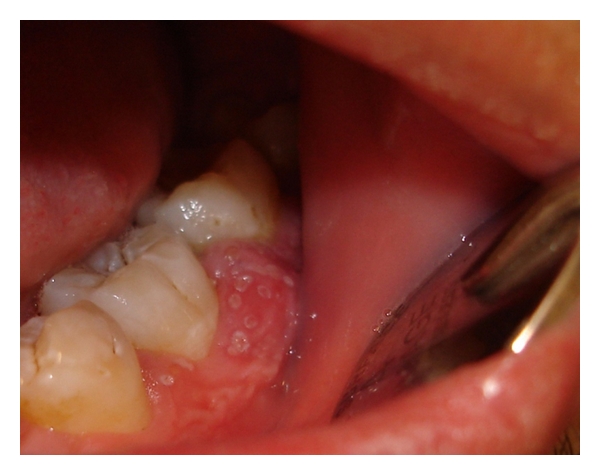
Clinical view of the periodontal abscess of the gingiva buccal attached to the lower left first molar and involving the adjacent papillae.

A 19-year-old patient was referred for evaluation of a painful swelling on the buccal aspect of the gingiva of the mandibular left first molar. The patient reported becoming aware of the swelling approximately two days prior to his arrival to the clinic. The swelling was accompanied by white ulcers the size of pinheads (Figure 1). His medical history was unremarkable, and he was free of systemic symptoms (e.g., lymphadenitis, malaise, fever, or skin lesions). He had recently undergone initial preparations for planned orthodontic treatment for crowding.

**Figure 2:**
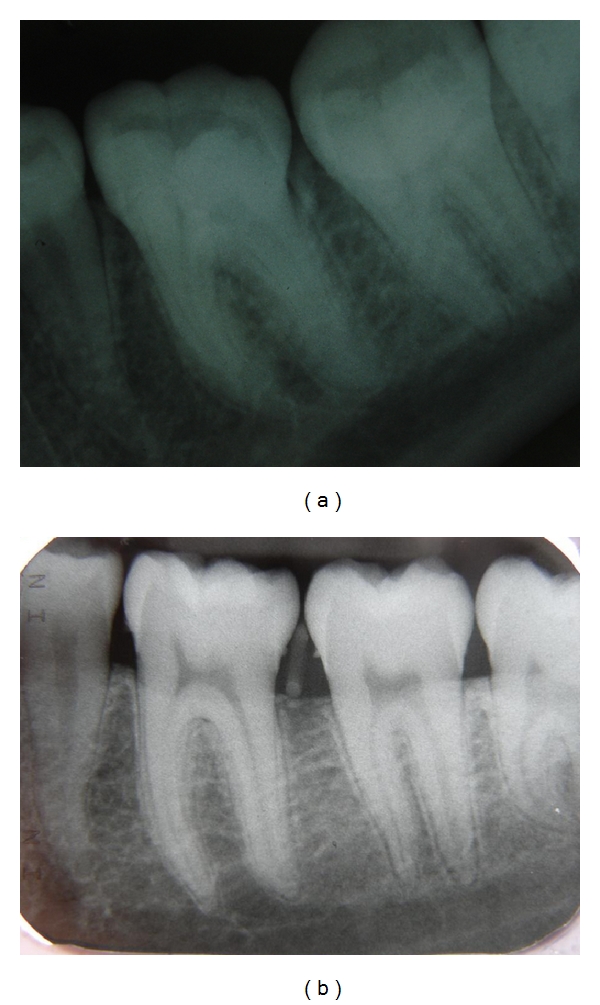
(a) Radiographic view of the elastic rubber band between the first and second lower left molars. (b) A proper periapical X-ray revealing an elastic band in the periodontal space.

**Figure 3:**
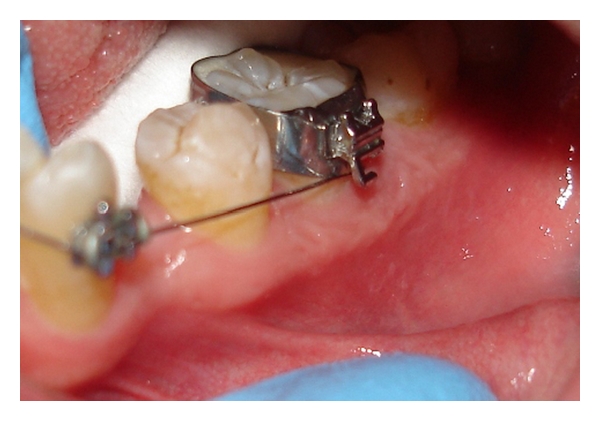
Clinical features at the one-month posttreatment follow-up with no clinical demonstration of residual pathology.

**Figure 4:**
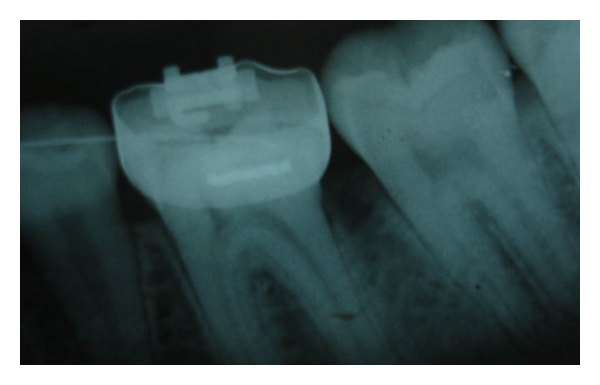
Radiographic view one month following treatment demonstrating damage to the alveolar bone between the first and second lower left molars.

The first X-ray was not orthoradial, and it revealed a small ill-defined radio-opaque area on the mesial aspect of the interproximal alveolar crest (Figure 2(a)). An additional X-ray from an orthoradial angle clearly displayed the interproximal area in which a radio-opaque, rectangular-shaped mass was discernable, as was subgingival calculus (Figure 2(b)). The elastic rubber band was removed by periodontal curettage. The clinical appearance at the one-month follow-up indicated complete recovery of the soft tissue (Figure 3), but the radiographic view revealed residual alveolar bone loss (Figure 4).

## 3. Discussion

The present report emphasizes the need for appropriate imaging to diagnose pathological conditions of the periodontium. It also highlights potential risks to the periodontium caused by using orthodontic elastic bands. Localized periodontitis and periodontal abscesses can be associated with a variety of dental material, such as silicone impression materials [10, 11], rubber dam [12], and even self-inflicted gingival injury due to habitual fingernail biting [13]. Localized reactive overgrowths of the gingiva can include the differential diagnoses of pyogenic granuloma [14–17], peripheral giant cell granuloma [14, 17], and periodontal abscess [17]. They can result from the invasion of pyogenic bacteria through the pocket epithelium, secondary to microtrauma or blockage of flow of inflammatory exudates from within the periodontal pocket. Entrapment of foreign bodies may serve as a trigger for these events [17, 18]. Several millimeters of periodontal attachment and alveolar bone can be lost within as little as a few days. The onset is sudden and accompanied by an acute inflammatory response (purulence) during which tissue necrosis takes place [17]. A painful gingival swelling may occur anywhere around the affected teeth. Swelling might involve the vestibule or cheek, since pus follows the path of least resistance. Depending on the severity of the infection, the patient may experience regional lymphadenitis, malaise, or fever. Such circumstances can represent a true emergency situation [17].

 Foreign material may cause and aggravate gingival lesions [2, 18]. A foreign body might induce both inflammatory and noninflammatory gingival changes manifested clinically as swelling and/or discoloration [2]. Koppang et al. [2] found that the mandibular and maxillary posterior segments were most frequently affected with foreign body gingival lesions (34% and 29%, resp.), followed by the maxillary anterior region (26%) [2]. They commented that these findings are probably attributable to the high frequency of dental procedures in these segments. Elastic bands should not be used on crowns of teeth without provision for stabilization [3]. A rubber band that slips undetected under the gingiva might move along the roots, resulting in significant loss of alveolar bone [3].

Foreign body-induced reaction should be included in the differential diagnosis of gingival overgrowths. Periodontal abnormalities occurring when orthodontic elastic separators are used should raise the possibility of a band impinging into the biological width. Appropriate imaging is essential for accurate diagnosis, especially when those devices are radiopaque.
